# Low-Intensity Pulsed Ultrasound Treatment for Non-unions of Long Bone Fractures in a Scottish District General Hospital

**DOI:** 10.7759/cureus.34159

**Published:** 2023-01-24

**Authors:** Petra Haller, Perrico Nunag, Antonios Papadopoulos

**Affiliations:** 1 Department of Orthopaedics, Dr Gray's Hospital, Elgin, GBR; 2 Department of Orthopaedics, Dr Gray’s Hospital, Elgin, GBR

**Keywords:** low-intensity pulsed ultrasound (lipus), covid-19, fracture healing, fracture non-union, exogen™

## Abstract

Objective: Despite advances in treatment, the management of fracture non-union remains a challenging and complex problem in orthopaedics. Low-intensity pulsed ultrasound (LIPUS) treatment has been shown to be an effective, non-invasive, affordable treatment option. This treatment was evaluated in a Scottish district hospital over a nine-year period, which included the COVID-19 pandemic.

Materials and methods: This submission describes a case series at Dr Gray’s Hospital in Scotland, 18 patients in whom fracture non-union was treated using LIPUS.

Results: An overall healing rate of 94% was achieved. Exogen™ (Bioventus LLC, NC, USA) proved to be most successful in oligotrophic non-union. No observed patient demographic appeared predictive of outcome. LIPUS treatment failed in one case. No significant adverse effects of LIPUS were detected.

Conclusion: LIPUS represents a useful, cost-effective potential alternative to revision surgery. LIPUS may therefore be the preferred treatment when surgical intervention and face-to-face interactions are to be minimised, as during the COVID-19 pandemic.

## Introduction

Optimum treatment of non-union following fractures remains a challenging problem. The current gold standard management, open reduction and internal fixation with bone graft, carries a significant socio-economic burden [1] and necessitates lengthy patient and health service engagement. Kanakaris and Giannoudis estimate the direct costs to be £7,000-£79,000 per non-union [2]; however, this might represent only 6.7%-17.2% of the overall treatment cost [3]. Some estimates of indirect costs, arising mainly from loss of productivity [1,3] and primarily borne by the patient, are as much as 82.8%-93.3% of the overall cost of treatment [3].

The incidence of fracture is estimated at 3.6 fractures/100 people/year in the UK [4]. Literature review suggests that non-union is diagnosed in 5%-10% [5]. A recent Scottish epidemiological study identified 4,895 cases of non-union requiring operative treatment in the five-year period from 2005 to 2010 [6]. Therefore, the direct cost of treating non-union in Scotland alone, based on estimates quoted above [2], is likely to be in the order of millions of pounds per year.

The COVID-19 pandemic created its own unique challenges in this already complex situation, with hospital beds at a premium, delays to non-emergency surgery and mandatory avoidance of face-to-face intervention [7]. After two years under such conditions, the advantages of non-operative management became abundantly clear: low-intensity pulsed ultrasound (LIPUS) is an effective, non-invasive, accessible treatment for non-union [8], which can be self-administered in the patient’s own homes.

Therapeutic low-intensity pulsed ultrasound (LIPUS) delivers micro-mechanical stress [9] to the tissues, causing nano-stimulation of the bone and resulting in increased growth factor production, increased expression of osteogenic genes through a COX2- cascade [10], osteoblastic differentiation, mesenchymal cell presence and vascularisation at the fracture site [5,11]. This is thought to lead to improved bone healing, which is critical in disturbed biology [11,12]. LIPUS devices have long been in commercial use, with encouraging results [13]. Historic analysis of a cohort of 767 patients with chronic non-union (non-union persisting in excess of one year) from the US Exogen™ Registry demonstrated a healing rate of 86.2% [13].

The exact definition of fracture non-union varies widely within the orthopaedic community [14]. In common with previously published large studies [15,16], this study used a definition of fracture non-union based on guidelines issued by the National Institute for Health and Care Excellence (NICE) in 2018 [9]. Radiological non-union for long bones was defined as either a fracture still present at least six months from initial injury or fixation, or a fracture characterized by failure of callus formation as evidenced by at least two consecutive radiographs. In the case of conservatively treated smaller bones, such as metatarsals and metacarpals, non-union was defined as a lack of satisfactory radiological union or failure of radiological progression on at least two consecutive radiographs within three months of initial injury or fixation.

In this retrospective study, Exogen™ (Bioventus LLC, NC, USA) was used to treat a variety of cases of fracture non-union at a district hospital in a remote, rural area of Scotland, in 18 patients who underwent LIPUS during the nine-year period from 2013 to 2021.

## Materials and methods

Patients were identified retrospectively from the local Exogen™ Order Registry between 2013 and 2021. The inclusion criteria were persistent pain at the fracture site and either absence of radiological union or no progression of callus on two consecutive radiographs at six months (in the case of long bone fracture) or three months (in the case of small bone fracture) from the date of injury or initial fixation [9].

All patients were managed by consultant orthopaedic surgeons at Dr Gray’s Hospital. The decision to offer LIPUS was individualized in this series. During the COVID-19 pandemic, remote consultations were employed to comply with restrictions. LIPUS was offered to patients with persistent pain and absence of callus progression on radiographs in accordance with the criteria. Patients gave informed consent for the treatment with LIPUS on the understanding that surgical intervention would be offered should LIPUS fail to generate adequate bone healing.

LIPUS stimulation employed Exogen 150™ (formerly Exogen Express™) equipment. The intended treatment area was delineated in ink by the orthopaedic consultant. All patients received adequate training and were deemed competent to self-administer low-intensity pulsed ultrasound stimulation via a transducer held over the delineated area and fixed in place using a strap, using a coupling gel to aid acoustic conduction. The transducer delivered acoustic radiation with a temporal average power of 30 milliwatts/cm^2^, operating frequency of 1.5 MHz, pulse width of 200 microseconds and repetition rate of 1 KHz, as described by NICE [9]. Treatment plans consisted of daily sessions of 20 minutes, continued for a minimum of 60 doses.

Clinical and radiological progress was assessed by the consultant orthopaedic surgeon at regular intervals.

The primary outcome measure was fracture healing, as defined by the complete resolution of clinical symptoms including pain, accompanied by satisfactory radiological union leading to discharge from the fracture clinic with no requirement for further follow-up.

The outcome was deemed successful or unsuccessful on the basis of both clinic letters and radiographs recorded after treatment with LIPUS. The outcome was evaluated at the final outpatient visit prior to discharge from the clinic (in the case of the full resolution of symptoms and satisfactory radiographic appearance) or when a decision was made to offer subsequent operative fixation.

Discharged patients were given self-awareness instructions and offered an open return to the Outpatient Department.

## Results

Eighteen patients with fracture non-union were identified from the registry from 2013 to 2021, and all were included in the study.

**Figure 1:**
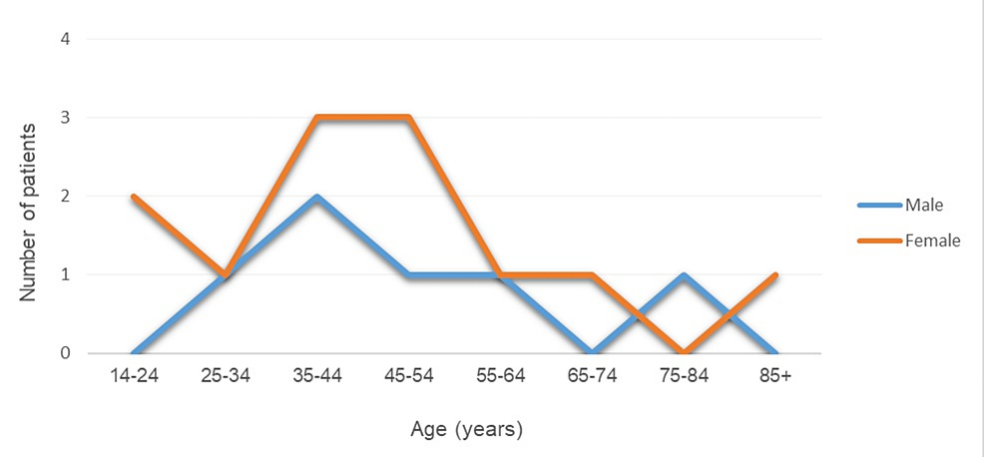
Age distribution of patients

The patient group comprised six males and 12 females. The average age at the time of injury was 46.6 years (range: 14-88 years). The average age did not differ significantly between males (48.3 years) and females (45.8 years). The age distribution is shown in Figure 1.

Five patients underwent operative fixation prior to LIPUS therapy, and 13 patients were managed conservatively throughout. A wide range of fracture types was represented in the study, of which 15 developed oligotrophic non-union and three developed hypertrophic non-union, as shown in Table 1.

**Table 1:** Demographic data, non-union classification, treatment times and outcomes TM: trademark, M: male, F: female, ORIF: open reduction and internal fixation, CONS: conservative treatment, CT: computed tomography, MR: magnetic resonance imaging, MC: metacarpal bone, MT: metatarsal bone, n/a: not applicable

Patient	Age	Gender	Anatomical location	Fracture pattern	Initial treatment	Type of non-union	Imaging modality confirming non-union	Time to Exogen™ (months)	Length of Exogen™ treatment (months)	Outcome
1	50	M	Femoral shaft	Comminuted metaphyseal with intercondylar progression	ORIF	Oligotrophic	CT	10.9	5.5	Healed
2	48	F	Lateral malleolus	Weber B	CONS	Oligotrophic	MR	18.3	5	Healed
3	27	M	Radius and ulna	Displaced, midshaft diaphyseal	ORIF	Hypertrophic	X-ray	8.2	6.7	Healed
4	68	F	Tibia	Comminuted, distal third diaphyseal	ORIF	Oligotrophic	X-ray	6.2	4.9	Healed
5	57	F	Lateral malleolus	Weber B	CONS	Hypertrophic	CT	11.4	4.9	Healed
6	76	M	Lateral malleolus	Weber B	CONS	Oligotrophic	CT	7.5	2.8	Healed
7	24	F	Third and fourth metacarpus	Comminuted, oblique diaphyseal with butterfly segment in the third MC	CONS	Oligotrophic	X-ray	4	3.2	Healed
8	41	M	Clavicle	Comminuted midshaft	CONS	Oligotrophic	CT	5.4	n/a	Lost to follow-up
9	42	F	Lateral malleolus	Weber B	CONS	Oligotrophic	X-ray	2.4	5	Healed
10	51	F	Clavicle	Midshaft oblique	ORIF	Oligotrophic	X-ray	6.7	3.2	Healed
11	35	F	Lateral malleolus	Weber B	CONS	Oligotrophic	X-ray	3.7	2.4	Healed
12	31	F	Fifth metatarsus	Jones	CONS	Oligotrophic	X-ray	3	2.8	Healed
13	43	F	Fourth metatarsus	Proximal diaphyseal stress	CONS	Hypertrophic	X-ray	3.3	n/a	Exogen™ failed
14	14	F	Fifth metatarsus	Jones	CONS	Oligotrophic	X-ray	6.5	7.3	Healed
15	88	F	Femoral neck	Pertrochanteric	ORIF	Oligotrophic	X-ray	6.8	4.6	Healed
16	38	M	Fifth metatarsus	Jones	CONS	Oligotrophic	X-ray	3.6	3.7	Healed
17	49	F	Fifth metatarsus	Base of the fifth MT	CONS	Oligotrophic	X-ray	6.9	2.8	Healed
18	58	M	Fifth metatarsus	Jones	CONS	Oligotrophic	X-ray	4.2	2.1	Healed

Available data were reviewed for the presence of comorbidities considered to be associated with failure of union post-fracture [12,17], the most commonly cited being smoking, alcoholism, diabetes, high body mass index (BMI) and intrinsic bone disorder [17]. Relevant patient comorbidities are summarized in Table 2.

**Table 2:** Observed patient-related risk factors BMI: body mass index, M: male, F: female

Patient	Gender	Smoking	Alcohol Excess	Comorbidities	BMI
1	M	No	Unknown	No	23
2	F	No	No	No	38
3	M	No	No	No	27
4	F	No	Social drinker	No	Unknown
5	F	No	No	No	39
6	M	Ex	No	Type 2 diabetes mellitus	29
7	F	No	No	No	24
8	M	Ex	No	No	Unknown
9	F	No	No	No	28
10	F	Heavy	No	No	23
11	F	Ex	Social drinker	Type 1 diabetes mellitus, hyperthyroidism	40
12	F	No	Social drinker	No	24
13	F	No	No	No	45
14	F	No	No	No	Unknown
15	F	Ex	Social drinker	Osteoporosis	27
16	M	Ex	No	Post-transplant diabetes, immunosuppression	28
17	F	No	No	No	26
18	M	Ex	Yes	No	31

Smoker or ex-smoker status was documented in 39% of patients and diabetes or other relevant comorbidities in 22%. Of our cases, 28% declared themselves non-teetotal; however, only patient (6%) one was identified as drinking alcohol to excess. The average BMI (for the 15 patients for whom this data was available) was 30, with 27% being in the healthy range. Overall, no risk factors were identified in six out of the 18 patients, while the remaining 67% of patients had one or more risk factors.

The Exogen 150™ or Exogen Express™ machine was fitted on an average of 6.6 months after the index injury or fixation (range: 74-557 days). All patients bar one reported compliance with the planned treatment regime, which lasted between 2.1 months and 7.3 months (average duration of 4.2 months or 127 days). Patient 8 demonstrated significant initial improvement with LIPUS treatment and became symptom-free, before ceasing to attend further follow-ups. (This patient was included in the overall healing rate, but their data was excluded from the length of treatment analysis.)

**Figure 2:**
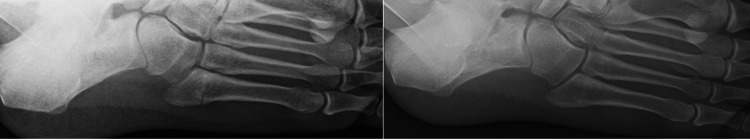
Pre- and post-treatment radiographs

**Figure 3:**
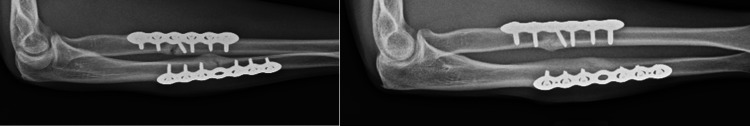
Pre- and post-treatment radiographs

Clinical and radiological follow-up at the Outpatient Department by the consultant orthopaedic surgeon continued throughout treatment, with reviews occurring every nine weeks on average (Figure 2 and Figure 3). Patient feedback regarding LIPUS was very positive.

Treatment with Exogen™ was successful in 17 out of 18 cases. Three patients reported initial increase in pain during the first week of Exogen™ treatment, which quickly settled. No other complications were reported. There were no late failures in successfully treated patients, and no patient required re-admission in respect of the same fractured bone. The overall healing rate was therefore 94%.

Exogen™ treatment was unsuccessful in one patient, who underwent ORIF with bone grafting two years after the index injury of the fourth metatarsal stress fracture. Apart from a BMI of 45 kg/m^2^ (the highest recorded BMI in our series), this patient had no other characteristics predictive of failure of union after fracture [17], unless the presence of a stress fracture was indicative of a pre-existing bone disorder.

Our case series of 18 fracture non-unions spanned 10 different bones, including lateral malleolar fracture (five cases), fifth metatarsal fracture (five cases), clavicle fracture (two cases), fourth metatarsal stress fracture, femoral shaft fracture, femoral neck fracture, tibial fracture, combined radius and ulna fracture, and combined third and fourth metacarpal fracture (one case each).

## Discussion

LIPUS has been used in the treatment of recalcitrant fracture non-union for many years. Several studies demonstrate the efficacy of LIPUS in this context [9,15,16].

A NICE meta-analysis evaluated the treatment of 1,441 cases of fracture non-union using LIPUS, suggesting an estimated healing rate of 82%. When the definition of non-union was restricted to fractures of at least eight months duration, the pooled estimate of effect size was 84% [9]. Overall, the estimated healing rate of 82% is reported in a review of pooled data for established fracture non-unions [9]. Our study, which yielded a healing rate of 94%, concurs with these findings. Furthermore, in line with published studies [8,9], no adverse effects were observed.

There are a number of documented patient-specific risk factors for the development of fracture non-union [12,17]. Zura et al. noted increased odds ratios for the development of non-union associated with male gender, smoking, obesity, excess alcohol intake and comorbidities such as diabetes and bone disorder [17]. None of these patient groups appeared to be significantly over-represented in our small case series.

Only one fracture non-union remained unresolved after treatment with LIPUS. This was the only stress fracture and one of only three cases of hypertrophic non-union in this case series.

As LIPUS can be self-administered at home, this alternative to operative fixation was particularly valuable during the COVID-19 pandemic. In the Scottish Highlands, where frequent travel to attend a face-to-face appointment is routinely inconvenient and costly, difficulties were exacerbated by limitations imposed by COVID-19 restrictions. LIPUS remained accessible and convenient both for patients and for the orthopaedic team despite the COVID-19 pandemic.

## Conclusions

No firm conclusions can be drawn from this study owing to the limitations imposed by its retrospective nature and the small number of participants. However, this study encompasses a wide variety of fracture types, and its results suggest that oligotrophic non-union may be particularly amenable to LIPUS treatment.

The healing rate of LIPUS treatment of fracture non-union in this study was 94%, concurring with larger published trials and comparable to outcomes after surgery. While the current waiting time for elective non-union surgery is excessive, LIPUS treatment is readily available and can be initiated soon after non-union has been diagnosed.

Although circumstances dictate minimal face-to-face contact with patients (as was the case during the COVID-19 pandemic), this study demonstrates that LIPUS presents a viable alternative to surgical fixation, which is acceptable to patients and may permit significant savings to be made in both direct and indirect costs.
